# Comparison of kidney-tonifying and blood-activating medicinal herbs vs NSAIDs in patients with knee osteoarthritis: A protocol for a systematic review and meta-analysis

**DOI:** 10.1097/MD.0000000000019370

**Published:** 2020-02-28

**Authors:** Hetao Huang, Sicong Huang, Guihong Liang, Lingfeng Zeng, Jianke Pan, Weiyi Yang, Hongyun Chen, Jun Liu, Biqi Pan

**Affiliations:** a Second School of Clinical Medicine, Guangzhou University of Chinese Medicine; b Department of Orthopaedics, Foshan Second People's Hospital; c Department of Orthopaedics, Second Affiliated Hospital of Guangzhou University of Chinese Medicine, Guangdong Provincial Hospital of Chinese Medicine; d Department of Traditional Chinese Medicine, GuangDong Women and Children Hospital, China.

**Keywords:** Chinese medicine, knee osteoarthritis, meta-analysis, randomized control trials

## Abstract

**Background:**

Knee osteoarthritis (KOA) is one of the most common chronic muscular diseases in old people. In recent years, people are more and more interested in the use of Chinese herbal medicine (CHM) in the treatment of KOA, such as kidney-tonifying and blood-activating medicinal herbs (KTBAMs) in the treatment of KOA. Many studies have confirmed that KTBAMs are effective in the treatment of KOA. However, it is still unknown whether KTBAMs and NSAIDs are more effective in the treatment of KOA. Therefore, we evaluated the efficacy and safety of KTBAMs and NSAIDs in the treatment of KOA.

**Methods:**

Randomized controlled trials (RCTs) from online databases including PubMed, Embase, the Cochrane Library, China National Knowledge Infrastructure, Chinese Scientific Journal Database, Wanfang Data, and Chinese Biomedical Literature Database that compared the efficacy of KTBAMs and NSAIDs in the treatment of KOA were retrieved. The main outcomes included the evaluation of functional outcomes, pain and adverse effects. The Cochrane risk of bias (ROB) tool was used to assess methodological quality.

**Results:**

The literature will provide a high-quality analysis of the current evidence supporting KTBAMs for KOA based on various comprehensive assessments including the total effective rate, visual analog scale scores, Western Ontario and McMaster Universities Osteoarthritis Index (WOMAC), Lequence scores, Knee Society Scale (KSS) scores, and adverse effects.

**Conclusion:**

This proposed systematic review will provide up-to-date evidence to assess the effect of KTBAMs in the treatment for patients with KOA.

**Research Registry registration number:**

: reviewregistry 783.

## Abstract 2

Supplemental Digital Content is available in the text

## 1 Introduction

Knee osteoarthritis (KOA) is one of the most common chronic muscular diseases in old people.^[1]^ The main manifestations of KOA are pain and dysfunction in the knees, which affect quality of life and lead to a high rate of disability in elderly individuals. The approximate prevalence of KOA in the general population throughout the world is 12% to 35%.^[2]^ KOA has a heavy socioeconomic burden in developed countries. Recently, KOA has become one of the global burden diseases. In some Asian countries, the high prevalence of KOA has increased medical care expenditures and attracted much government attention.^[3]^

The main objectives in the management of KOA have been to alleviate pain, educate patients about their disease, restore function, slow down the progression of disease and maintain a health-related quality of life.^[4]^ Traditionally, the management of end-stage KOA for relieving pain and improving function has been knee arthroplasty.^[1]^ Conservative approaches address early stages of the disease, such as oral NSAIDs, hyaluronic injection, and self-management, but the clinical results may not satisfy patients. In light of this situation, alternative treatments such as herbal preparations,^[5]^ acupuncture,^[6]^ moxibustion,^[7]^ massage,^[8]^ and Tai-chi^[9]^ have been investigated for their efficacy in randomized controlled trials (RCTs) and have drawn attention.

As an alternative therapy, Chinese herbal medicine (CHM) or herbal products have been used and recommended by many clinicians. These have been indicated to help alleviate KOA symptoms and reduce costs.^[10–13]^ Kidney-tonifying and blood-activating medicinal herbs (KTBAMs) are one type of Chinese herbal recipe consisting of herbals that can ‘tonify kidney’ and ‘activate blood’ based on traditional Chinese theory. Research on the mechanism of some recipes of KTBAMs have shown their effectiveness in promoting chondrocyte proliferation, inhibiting sodium nitroprussiate-induced chondrocyte apoptosis, and regulating the expression of vascular endothelial growth factor (VEGF) and hypoxia-inducible factor-1α (HIF-1α).^[14–18]^ Recently, researchers have reported that KTBAMs can help control KOA-related symptoms and have been widely used in many Asian countries.^[19]^ NSAIDs are the most popular medicine because of their promising effect for KOA, although they are accompanied by high costs and many related side effects.^[20]^ KTBAMs, alone or combined with conventional pharmaceutical drugs, has also been commonly used for the clinical management of KOA. Some researchers^[21–22]^ have published systematic reviews of the efficacy and safety of traditional Chinese medicine prescriptions in the treatment of KOA. However, most of the systematic reviews have been based on intervention measures that include “traditional Chinese medicine”, but there is no systematic review of a specific kind of traditional Chinese medicine. In particular, no study has systematically examined the effectiveness and safety of KTBAMs for KOA according to the Preferred Reporting Items for Systematic reviews and Meta-Analyses (PRISMA) until now. To assist clinical practice and possibly to reduce the heavy burden of KOA patients, it is important to systematically review the current evidence of KTBAMs compared with NSAIDS. Thus, we performed a meta-analysis of RCTs to assess the evidence for the efficacy and safety of KTBAMs for KOA in comparison with NSAIDs.

In the previous systematic reviews of the efficacy and safety of traditional Chinese medicine prescriptions in the treatment of KOA, the included studies compared different prescriptions with different efficacies and mechanisms of action, and there was a high level of clinical heterogeneity among the studies.^[23–24]^ In contrast, in the present study, the interventions were strictly limited to KTBAMs, and the control measures were limited to NSAIDs. To some extent, the bias caused by heterogeneous sources and drugs with different mechanisms of action was reduced, and the results of this study have higher clinical significance. In addition, our study incorporated additional and updated clinical research reports, which complemented and updated the previous systematic reviews. In conclusion, it is necessary to study the efficacy and safety of KTBAMs in the treatment of KOA.

## 2 Methods

### 2.1 Data sources and search strategy

The study was approved by the ethics committee of Guangdong Provincial Hospital of Chinese Medicine. We will adhere to the Preferred Reporting Items for Systematic Reviews and Meta-analysis (PRISMA) statements for reporting systematic reviews. Seven databases, including PubMed, EMBASE, Cochrane Central Register of Controlled Trials, China National Knowledge Infrastructure, Chinese Scientific Journal Database, Wanfang Data, and Chinese Biomedical Literature Database, were investigated from their inception through December 2019. The reference lists of retrieved papers were also studied. The following search terms were used individually or in combination. The mesh terms in this paper are as follows: ‘osteoarthritis, knee’, ‘Anti-Inflammatory Agents, Non-Steroidal’, and the entry terms are as follows: ‘Bushen’, ‘Kidney-tonifying’, ‘Blood-activating’, ‘arthritis’, ‘osteoarthritis’, ‘knee osteoarthritis’, ‘knee arthritis’, and ‘osteoarthritis of knee joint’. To increase the search range, no date and no language limits were imposed. Additionally, no restrictions on population characteristics were imposed. The specific search strategies for PubMed are shown in the Supplemental Table.

### 2.2 Inclusion criteria and study selection

#### 2.2.1 Participants

Only published articles enrolling adult participants with a diagnosis of KOA will be included. The patient's gender, age, and grades of KOA will not be limited.

#### 2.2.2 Interventions

The intervention group will have treated with KTBAMs (the traditional Chinese medicine prescription must have contained both a recognized CHM with kidney-tonifying effects and a CHM with blood-activating effects).

#### 2.2.3 Comparisons

The control group will have received NSAIDs alone.

#### 2.2.4 Outcomes

The primary outcomes of this meta-analysis were “total effective rate”, and the secondary outcomes were “VAS scores”, “WOMAC scores”, “Lequence scores”, “KSS scores”, and “adverse effects”.

#### 2.2.5 Study design

**Figure 1:**
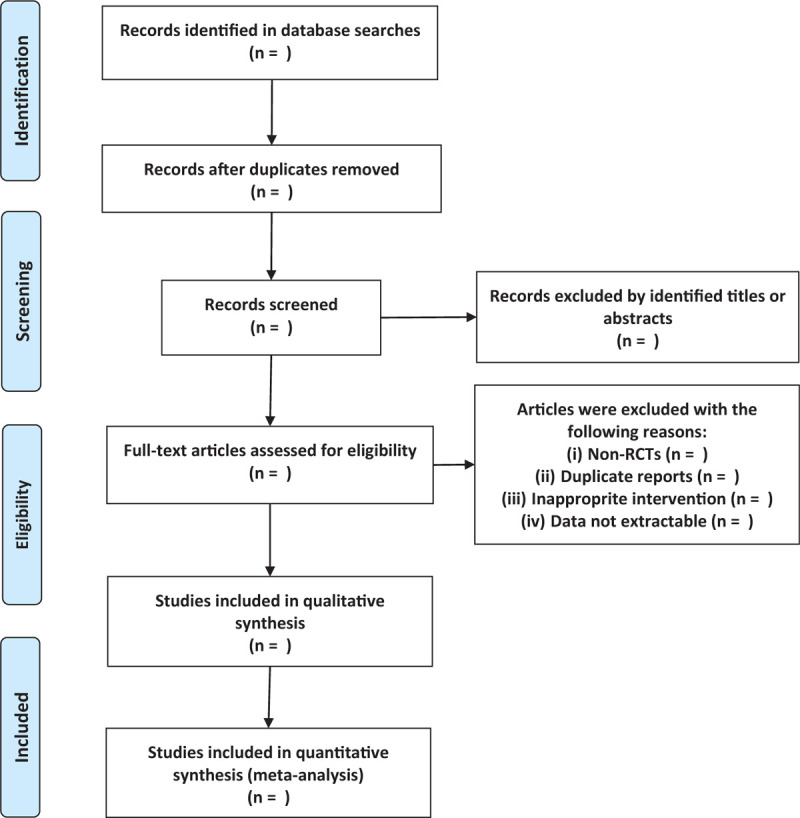
Flow diagram of study selection.

RCTs will be considered eligible for our study. Articles will be excluded if they are case reports, letters, editorials, and nonhuman studies. The flow diagram of the study selection is shown in Fig. 1.

### 2.3 Data extraction

Data extraction included the first author's name, year of publication, sample size, diagnostic criteria, age and sex of the participants, details of the intervention and control conditions, treatment duration, and outcome measurements for each study. Two authors (HTH, SCH) independently conducted the data extraction according to predefined criteria. Any uncertainty was resolved through discussion with another author (BQP). The reasons for exclusion were recorded. The data were extracted from the included RCTs to a predefined Excel table (Microsoft Corp, Redmond, WA) and cross-checked by the two reviewers (HTH, GHL). In the event of missing data, we will attempt to contact the corresponding authors for details.

### 2.4 Assessment of methodologic quality

Two authors (HTH, LFZ) independently assessed the methodological quality of each trial according to the standards advised by the Cochrane Handbook.^[25]^ Disagreements, if any, were resolved by discussion and reached consensus through a third reviewer (BQP). The risk of bias (ROB) was evaluated for each study by assessing the randomization process, the treatment allocation concealment, blinding of participants and personnel, blinding of outcome assessment, the completeness of the data, the reporting of results, and other biases. Selective reporting bias was judged according to the published protocols for the registered clinical trials that were contained on the Chinese clinical trial registry (http://www.chictr.org) and international clinical trial registry of the US National Institutes of Health (http://clinicaltrials.gov) websites. We compared the outcome measures between the study protocol and the final published trial.

### 2.5 Data analysis

Data analysis was carried out using Review Manager software (V.5.3) provided by the Cochrane Collaboration. Given the characteristics of the extracted data in the review, continuous outcomes were expressed as the mean differences (MDs) with 95% confidence intervals (CIs). Differences in categorical variables were expressed as risk ratio (RR) values and 95% CIs. Heterogeneity was assessed by means of *I*^2^ statistics. *I*^2^ ≥ 50% represented high heterogeneity. A standardized mean difference (SMD) was used when the studies included in the meta-analysis assessed the outcome based on different scales (e.g., visual analog scale (VAS) 0–10 and VAS 0–100). Initially, a fixed-effect model would be used to compare the outcomes, unless the heterogeneity tests indicated that the *I*^2^ statistic ≥50% and substantial heterogeneity existed between studies; in this case, the reasons for this heterogeneity would be searched for and a random-effect model would be used for comparison. The subgroup analysis was undertaken according to prespecified criteria to investigate heterogeneous results or to determine the effect of prespecified criteria on the pooled estimate. We assumed that clinical differences would mainly originate from the treatment duration and the dosage and frequency of NSAIDs; therefore, subgroups were divided based on these factors. Publication bias was analyzed by funnel plot analysis if sufficient studies (n ≥ 10) were found.

### 2.6 GRADE the evidence

The GRADE system was used to evaluate the quality of the evidence for each outcome. GRADE-pro GDT Online Tools (available on https://gradepro.org/) were used to evaluate the evidence regarding the included outcomes. Initially, RCTs were considered to be of high confidence in estimating an effect, and observational studies were considered to be of low confidence in estimating an effect. The reasons that may decrease the level of confidence included ROB, inconsistency, indirectness, imprecision, and publication bias. The reasons that may increase the level of confidence included a large effect, dose response, and accounting for all plausible residual confounding and bias. The GRADE evidence was divided into the following categories:

(1) high-quality evidence, which indicated that further research was unlikely to change the confidence in the estimate of the effect;
(2) moderate-quality evidence, which indicated that further research was likely to have an important impact on the confidence in the estimate of the effect and may change the estimate;
(3) low-quality evidence, which indicated that further research was likely to have an important impact on confidence in the estimate of the effect and was likely to change the estimate; and
(4) very low-quality evidence, which indicated that we were very uncertain about the results.

## 3 Discussion

Traditional Chinese medicine has been widely used in clinical practice in China as an alternative approach for KOA. Previous studies have demonstrated the efficacy of herbal medicines, such as Duhuo Jisheng decoction (DJD).^[26,27]^ Based on traditional Chinese medicine theory, tonifying kidney and activating blood is one of the most common approaches for KOA. DJD is a formula for the treatment of arthralgia and functional disorders by tonifying kidney and eliminating dampness. Our study will compare the efficacy and safety of KTBAMs and NSAIDs for KOA from RCTs.

The previous research supposed that the efficacy of NSAIDs was significantly more stable and reliable than that of KTBAMs (with respect to Western Ontario and McMaster Universities Osteoarthritis Index (WOMAC) scores, Lequence functional index scores and Knee Society Scale (KSS) scores). The reason why NSAIDs were more reliable may be due to the possibility that KTBAMs may exert their effects via several probable mechanisms from the pharmacodynamic point of view,^[19]^ and these mechanisms might have influenced drug concentrations and the subsequent drug effects. As an ancient traditional treatment, KTBAM therapy has developed over thousands of years in China. In the earliest published Chinese medical work, “Inner Classic of the Yellow Emperor” (475 B.C.–221 B.C.), KTBAM therapy was frequently reported as having beneficial outcomes. Despite the lack of knowledge about the biological mechanisms by which Chinese herbal therapy works for KOA, the multitarget therapeutic effect of traditional Chinese medicine has been recognized by many researchers. Some animal experimental studies have indicated that Chinese herbs decreased the levels of nitric oxide in the serum, synovium, and joint cartilage in osteoarthritic rabbits.^[28]^ Another study showed that Du-Huo-Ji-Sheng decoction (a KTBAM compound) exerted significant therapeutic effects in osteoarthritic rabbits, probably through inhibiting the expression of VEGF and hypoxia-inducible factor-1α.^[15]^ Yaotongning capsules (a KTBAM compound) promoted proliferation and glycosaminoglycan synthesis in IL-1β-induced chondrocytes and may have potential activity in treating chondrocyte degeneration caused by osteoarthritis.^[29,30]^ A study has found that kidney-tonifying and blood-activating Chinese herbs may suppress the expression of interferon regulatory factor 7 (IRF-7) by regulating the TLR4/MyD88 signaling pathway, resulting in less secretion of interleukin 6 (IL-6) and matrix metallopeptidase 13 (MMP-13), which alleviates inflammation and delays cartilage destruction.^[31]^ Another study reported the effects of low, medium and high doses of Bushen Huoxue recipe on knee arthritis in rats. It had been found that the pathological changes of knee arthritis in the low-, medium- and high-dosage groups were gradually alleviated, although the detailed mechanism of action was unknown.^[32]^

As the systematic review is based on the secondary research of published literature, there are undeniable methodological defects. In addition, the quality of the included studies determines the quality level and reliability of the final results. We will begin to conduct the review when the necessary trials are met, and all operating procedures will be performed in accordance of Cochrane Handbook to ensure that the provided information is helpful for clinicians and patients. This study is registered with the Research Registry and the unique identifying number is: researchregistry783 (https://www.researchregistry.com/browse-the-registry#registryofsystematicreviewsmeta-analyses/?view_13_search=reviewregistry783&view_13_page=1).

## Acknowledgments

We would like to thank Professor Holger Schulenemann, Chairman of GRADE Working Group, Department of Clinical Epidemiology and Biomedical Statistics, McMaster University, Canada; Professor Li Youping, Director of Cochrane Center in China; Professor Yang Kehu, Director of GRADE Center in China; Professor Tian Jinhui, Evidence-based Medicine Center of Lanzhou University for their training on Cochrane system evaluation and grade system knowledge.

## Author contributions

**Conceptualization:** Hetao Huang, Biqi Pan.

**Data curation:** Hetao Huang, Sicong Huang, Guihong Liang, Lingfeng Zeng.

**Formal analysis:** Hetao Huang, Sicong Huang, Lingfeng Zeng.

**Funding acquisition:** Jianke Pan, Weiyi Yang, Hongyun Chen.

**Investigation:** Hetao Huang, Jun Liu.

**Methodology:** Guihong Liang, Lingfeng Zeng, Jianke Pan.

**Project administration:** Hetao Huang, Sicong Huang, Guihong Liang, Biqi Pan.

**Resources:** Jianke Pan, Weiyi Yang, Hongyun Chen.

**Software:** Hetao Huang, Sicong Huang, Jianke Pan.

**Supervision:** Jianke Pan, Weiyi Yang.

**Validation:** Weiyi Yang, Biqi Pan.

**Visualization:** Jianke Pan.

**Writing – original draft:** Hetao Huang, Sicong Huang.

**Writing – review & editing:** Biqi Pan.

## Supplementary Material

Supplemental Digital Content
