# Supplementary material for: Comparison of kidney-tonifying and blood-activating medicinal herbs vs NSAIDs in patients with knee osteoarthritis: A protocol for a systematic review and meta-analysis
Source: Medicine (Baltimore). 2020 Feb 28;99(9):e19370. doi: 10.1097/MD.0000000000019370 (PMC7478677; doi:10.1097/MD.0000000000019370)

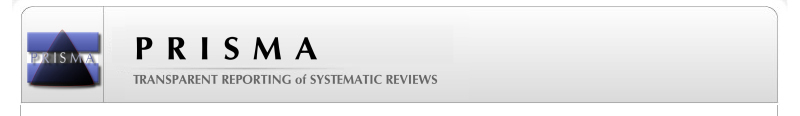
**PRISMA 2009 Flow Diagram**

**Screening**

**Included**

**Eligibility**

**Identification**

**Records identified in database searches
(n = )**

**Records after duplicates removed
(n = )**

**Records screened
(n = )**

**Records excluded by identified titles or abstracts
(n = )**

**Full-text articles assessed for eligibility
(n = )**

**Articles were excluded with the following reasons:**

1. **Non-RCTs (n = )**
2. **Duplicate reports (n = )**
3. **Inapproprite intervention (n = )**
4. **Data not extractable (n = )**

**Studies included in qualitative synthesis
(n = )**

**Studies included in quantitative synthesis (meta-analysis)
(n = )**

**Search strategies in Pubmed**

#1 Search (((((((((((((bu shen） OR reinforcing kidney） OR tonifying kidney） OR nourishing kidney） OR invigorate kidney） OR promote the function of kidney） OR impotence kidney） OR tonify the kidney） OR enrich the kidney） OR warm the kidney） OR supplement the kidney））

#2 Search (((((((((((((activating blood） OR activate blood circulation） OR promoting blood circulation） OR bloodactivating） OR huo xue） OR move blood） OR invigorate the circulation of blood） OR blood-activating） OR activate blood） OR promotes blood flow） OR activates blood） OR break blood） OR blood-breaking））

#3 #1 AND #2

#4 Search (((((((((osteoarthritis, knee[MeSH Terms]) OR Knee Osteoarthritides[Title/Abstract]) OR Knee Osteoarthritis[Title/Abstract]) OR Osteoarthritides, Knee[Title/Abstract]) OR Osteoarthritis Of Knee[Title/Abstract]) OR Knee, Osteoarthritis Of[Title/Abstract]) OR Knees, Osteoarthritis Of[Title/Abstract]) OR Osteoarthritis Of Knees[Title/Abstract]) OR KOA[Title/Abstract])

#5 # 3 AND #4


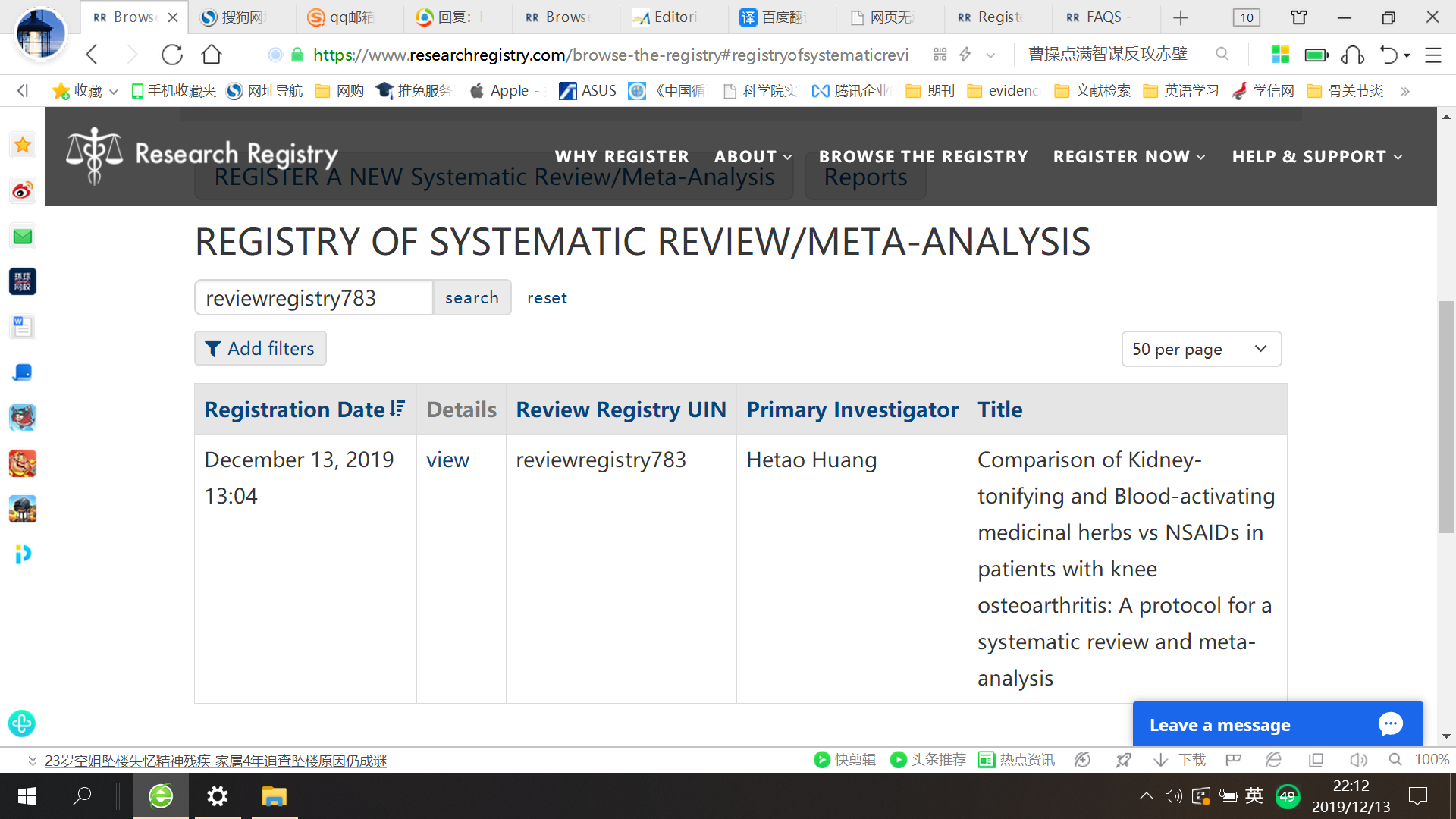

Supplement: Supplemental Digital Content [file medi-99-e19370-s001.doc]
